# Paeoniflorin Alleviates LPS-Induced Inflammation and Acute Myocardial Injury by Inhibiting PI3K/Akt/ERK-Mediated HIF-1α and TLR4/MyD88/NF-κB Inflammatory Signaling

**DOI:** 10.1155/mi/2346163

**Published:** 2025-11-19

**Authors:** Xiaowu Guo, Zhiguang Han, Jiahuan Sun, Shupeng Liu, Chuang Zhang, Gengrui Xu, Xiaodan Wang, Qiuhang Song, Hongxia Yang, Aiying Li

**Affiliations:** ^1^Department of Biochemistry and Molecular Biology, College of Pharmacy, Hebei University of Chinese Medicine, Shijiazhuang 050200, Hebei, China; ^2^Department of Epidemic Febrile Disease, College of Traditional Chinese Medicine, Hebei University of Chinese Medicine, Shijiazhuang 050200, Hebei, China; ^3^Hebei Key Laboratory of Chinese Medicine Research on Cardio-Cerebrovascular Disease, Shijiazhuang 050091, Hebei, China; ^4^Hebei Higher Education Institute Applied Technology Research Center on TCM Development and Industrialization, Shijiazhuang 050091, Hebei, China

**Keywords:** molecular docking, network pharmacology, paeoniflorin (PF), PI3K/Akt/ERK-mediated HIF-1α, sepsis-induced myocardial injury (SIMI), TLR4/MyD88/NF-κB-inflammation signaling

## Abstract

Sepsis-induced myocardial injury (SIMI) greatly increases the mortality rate of sepsis. Although paeoniflorin (PF) has been proven to improve survival in sepsis, the detailed mechanism of PF on SIMI remains elusive. In this study, network pharmacology revealed 90 overlapping targets between PF- and SIMI-related targets. Analysis using the molecular complex detection (MCODE) method identified a significant module with scores exceeding 30, comprising the top 10 targets: Akt1, STAT3, CASP3, BCL2, TP53, PTGS2, CXCL8, TLR4, CCL2, and ICAM1. These targets are involved in tissue repair during inflammatory response, apoptosis, immunity, and lipopolysaccharide (LPS) immune receptor activity. The enriched pathways in inflammatory signaling, include NF-κB signaling pathway, HIF-1 signaling pathway, MAPK signaling pathway, and PI3K-Akt signaling pathway. Molecular docking further verified the strong binding abilities of PF to PI3K, Akt1, ERK1, ERK2, HIF-1α, TLR4, and NF-κB. In LPS-induced sepsis rat model, PF pretreatment inhibited PI3K/Akt/ERK-mediated HIF-1α and TLR4/MyD88/NF-κB signaling, thereby reducing inflammation by decreasing the levels of tumor necrosis factor-α (TNF-α) and interleukin-1β (IL-1β) in serum and cardiac tissue. Ultimately, PF ameliorated SIMI by improving cardiac pathological and functional changes and mitigating myocardial injury markers, such as lactate dehydrogenase (LDH), CK-MB, cTnT/TNNT2, TNNI3/cTn-I, and aspartate aminotransferase (AST). Collectively, the PI3K/Akt/ERK-mediated HIF-1α and TLR4/MyD88/NF-κB inflammation signaling appear to be the primary mechanisms through which PF exerts its beneficial effects on SIMI.

## 1. Introduction

Sepsis is a leading cause of unacceptably high mortality rates worldwide [1]. One of its most severe complications is multiple organ dysfunction, which dramatically augments clinical mortality rates [2]. Among the various organs affected, the heart is particularly vulnerable, with studies showing that 40%–50% of sepsis patients suffer from myocardial injury [3]. Cardiac dysfunction is a life-threatening complication of sepsis [4], often serving as an early warning sign of a potentially fatal outcome, with a mortality rate reaching up to 70% [5]. Therefore, protecting the heart from damage is an effective therapeutic strategy in decreasing the mortality of sepsis.

Sepsis-induced myocardial injury (SIMI) is a severe consequence of excessive inflammatory response that arises from the complex interplay between the pathogen and the host [6]. The inflammatory storm, characterized by a systemic release of pro-inflammatory cytokines, is the core pathological mechanism driving sepsis [7]. The anti-inflammatory effects of the natural products, particularly those derived from Chinese herbs, have received a lot of attention globally in recent years. Paeoniflorin (PF), the primary active ingredient originated from the Chinese herb *Paeonia lactiflora*, has demonstrated potent anti-inflammatory effects in both in vitro and in vivo studies [8]. Promising research in experimental sepsis models has shown that PF can mitigate lipopolysaccharide (LPS)-caused organ injury, including heart, lung, and liver [9, 10], and improve sepsis survival by decreasing the production of inflammatory cytokines [11]. However, the detailed mechanisms underlying PF's protective effects against SIMI remain to be elucidated.

Network pharmacology is an emerging field that provides a comprehensive method to prompt the pharmacological mechanisms of drug ingredients by constructing drug-target–disease interaction networks. In the study, we performed network pharmacology to identify the targets of PF in alleviating SIMI. Additionally, we applied molecular docking to verify the binding affinities between PF and the core targets. Finally, based on the above analyses, we adopted LPS-induced sepsis rat model to demonstrate the pharmacological mechanisms of PF in treating SIMI.

## 2. Materials and Methods

### 2.1. Reagents

PF (purity 98.15%) was purchased from Shanxi TOSO Biotech Co., Ltd (China). Dexamethasone Sodium Phosphate Injection (Dex, cat: 2207082) was produced by Tianjin KingYork Group Hubei Tianyao Pharmaceutical Co., Ltd (China). A TLR4 inhibitor (TLR4-IN-C34-C2-COOH, C34, cat: HY-W092043) was obtained from MedChemExpress (USA). LPS (*Escherichia coli* 0111:B4) was purchased from Sigma–Aldrich (USA). Antibodies and ELISA kits in this study are detailed in Table 1.

### 2.2. Network Pharmacology

Protein targets of PF were identified from TCMSP (https://www.tcmsp-e.com/index.php), SwissTarget database (http://old.swisstargetprediction.ch/), TCMIP (http://www.tcmip.cn/TCMIP/index.php/Home/Login/login.html), GeneCards (https://www.genecards.org/), and bibliographic retrieval. The therapeutic targets of SIMI were predicted using GeneCards, OMIM (https://www.omim.org/), and DrugBank (https://go.drugbank.com/). The intersecting targets between PF and SIMI were collected using the VENNY 2.1 [12]. The intersecting targets were then analyzed using the STRING database (score > 0.7) to construct a protein–protein interaction (PPI) network, which was visualized using Cytoscape software 3.7.1 to identify the potential central targets of PF in treating SIMI [13]. The biological functions of PPI network were further clustered to obtain potential protein modules and TOP genes employing molecular complex detection (MCODE) method, a plug-in of Cytoscape software 3.10.3, with a score >4. Gene Ontology (GO) functional enrichment analysis and Kyoto Encyclopedia of Genes and Genomes (KEGG) pathway enrichment analysis were performed on the cross-targets using the DAVID database (DAVID, version 6.8, https://david.ncifcrf.gov/) and visualized applying an online tool (https://www.bioinformatics.com.cn/). Terms with a *p*-value < 0.05 and a fold enrichment >1.5 were considered significant.

### 2.3. Molecular Docking

Molecular docking was performed to evaluate the binding affinities between PF and the core targets received from the network pharmacology. First, the 2D structure of PF was achieved from the PubChem database (https://pubchem.ncbi.nlm.nih.gov/), converted to a 3D structure using Chem3D software, and saved in mol2 format. Then it was transformed into PDBQT format using Autodock Vina docking procedure (http://autodock.scripps.edu/). Second, high-resolution protein crystal structures of PI3K, Akt, ERK1, ERK2, HIF-1α, TLR4, and NF-κB were selected as the ligands. The PDB formats of these targets were downloaded from the RCSB PDB database (https://www.rcsb.org/), imported into PYMOL software to remove water molecules and heteromolecules. Subsequently, they were imported into Autodock Vina software to add hydrogen atoms and saved as pdbqt format. Finally, the docking boxes were formed between PF and the protein structures using Autodock Vina software. Binding energies below −5 kcal/mol were considered to indicate strong binding affinities. The results were saved with the lowest docking energy and were visualized using PYMOL software.

### 2.4. Experimental Animals and Protocols

Sprague–Dawley (SD) rats (7–8 weeks, weighing 220–250 g) were purchased from Beijing Charles River Laboratory Animal Technology Co., Ltd (China). All rats were placed in plastic cages maintained at 24 ± 2°C on a 12-h light/dark cycle for 1 week before experiments and were allowed free access to standard chow and water. All animal procedures were conducted according to the guidelines established by the Ethics Committee of Hebei University of Chinese Medicine (DWLL202203111, Shijiazhuang, Hebei, China).

**Figure 1:**
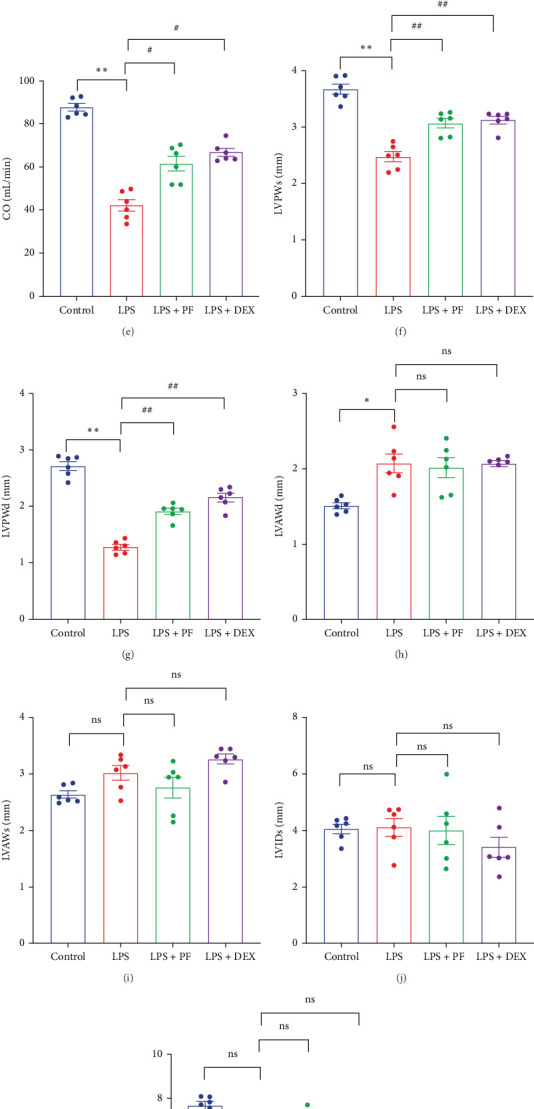
PF improved cardiac dysfunction in SIMI rats. (A) Representative images of ECG recorded by lead II in different groups. (B) Representative images by echocardiograph in each group. (C–K) The statistical analysis of EF, FS, CO, LVPWs, LVPWd, LVAWs, LVAWd, LVIDs, and LVIDd in each group (*n* = 6). (L) The flowchart of the study. Significance: *⁣*^*∗*^*p* < 0.05, *⁣*^*∗∗*^*p* < 0.01 vs. the control group; ^#^*p* < 0.05, ^##^*p* < 0.01 vs. the LPS group.

SD rats were randomly divided into four groups: control group (Control, *n* = 8), LPS group (LPS, *n* = 12), LPS–PF group (LPS + PF, *n* = 12), and LPS–Dex group (LPS + Dex, *n* = 12). Before LPS injection, LPS–PF and LPS–Dex groups were pretreated with PF at a dose of 60 mg/kg and Dex at a dose of 2 mg/kg for 3 days by intraperitoneal injection (i.p.), respectively. At the same time, the control and LPS groups received the same amount of saline in the same manner. LPS was injected intraperitoneally 2 h after the last drug administration in each group except the control group, which received the same amount of saline. To verify the inhibitory effect of PF on the TLR4 signaling pathway, its effect was compared with that of the TLR4 inhibitor C34 [14]. The study flowchart is presented in Figure 1L.

### 2.5. Electrocardiography (ECG)

ECG and Echocardiography were performed 6 h after LPS or saline administration. The rats were anesthetized with isoflurane, and ECG lead II data were recorded continuously for 60 s using the BL-420 s biological function experimental system (Techman Software, China).

### 2.6. Echocardiography

Echocardiography was performed to evaluate cardiac function using a Vevo 2100 system (Visual Sonics, Canada). The following parameters were evaluated: ejection fraction (EF), fractional shortening (FS), cardiac output (CO), LV systolic posterior wall thickness (LVPWs), LV diastolic posterior wall thickness (LVPWd), LV systolic anterior wall thickness (LVAWs), LV diastolic anterior wall thickness (LVAWd), left ventricular (LV) end-systolic internal diameter (LVIDs), and LV end-diastolic internal diameter (LVIDd). Vevo Lab 3.2.0 software was used to analyze the echocardiograms (FUJIFILM Visualsonics, Inc).

### 2.7. Examination for lactate dehydrogenase (LDH), CK-MB, cTnT/TNNT2, TNNI3/cTn-I, and aspartate aminotransferase (AST) in Serum

The rats were anesthetized with 3% pentobarbital sodium (5 mg/kg) via intraperitoneal injection. Serum was obtained by centrifugation after collecting blood from abdominal aorta. The levels of CK-MB, cTnT/TNNT2, and TNNI3/cTn-I were examined by ELISA kits. The levels of LDH and AST were detected using LDH activity assay kit and AST/GOT activity assay kit, respectively. LDH activity assay kit, AST/GOT activity assay kit, and ELISA kits for CK-MB, cTnT/TNNT2, and TNNI3/cTn-I are listed in Table 1.

### 2.8. Hematoxylin-Eosin (HE) Staining

Heart tissue samples were fixed in 4% paraformaldehyde, dehydrated, embedded, and cut into 4 μm thick sections. HE staining was employed to analyze the histopathological changes in cardiac tissue. The stained sections were observed and photographed using a light microscope (Leica DM4000B, Germany).

### 2.9. ELISA for Tumor Necrosis Factor-α (TNF-α) and Interleukin-1β (IL-1β) in Serum

Blood samples were collected from abdominal aorta, and the serum was gained by centrifugation at 1000 rpm for 15 min and stored at −20°C before analysis. The levels of TNF-α and IL-1β were detected using ELISA kits according to the manufacturer′s instructions. ELISA kits for TNF-α and IL-1β are shown in Table 1.

### 2.10. Western Blot

Protein was extracted from rat heart tissue using RIPA lysis solution containing PMSF, cocktail, and phosphorylase inhibitor. Protein concentration was evaluated using bicinchoninic acid (BCA) protein quantification kit (PC0020, Solarbio, China). Protein samples were separated on 10% SDS–PAGE gels and transferred to polyvinylidene fluoride (PVDF) membranes. The membranes were sealed with 5% skim milk for 90 min and incubated with primary antibodies overnight at 4°C. The primary antibodies used were: PI3K (1:2000), Akt (1:4000), P-Akt (1:4000), ERK (1:4000), P-ERK (1:4000), HIF-1α (1:1000), TLR4 (1:5000), MyD88 (1:1000), NF-κB (1:1000), and P-NF-κB (1:1000). GAPDH (1:10,000) or β-actin (1:10,000) was used as an internal control. The next day, the membranes were incubated with HRP-conjugated secondary antibodies (ZB-2301 or ZB-2305, 1:20,000, ZSGB). An ECL chemiluminescence kit (P10300A, NCM Biotech, China) and a chemiluminescence imaging system (OmegaLum W, USA) were applied to detect the targeted proteins. Image J software (NIH, USA) was used to analyze the gray values of the proteins. The antibodies are shown in Table 1.

### 2.11. Real-Time Fluorescent Quantitative PCR (qRT-PCR) Analysis

qRT-PCR was performed to analyze the levels of TNF-α and IL-1β in cardiac tissue using QuantStudio 1 real-time detection Instrument (Thermo Fisher Scientific, USA). Total RNA was extracted applying the total RNA kit II (R6934-01, Omega, USA), and its concentration and purity were measured. The RNA was reverse-transcribed into cDNA using PrimeScript RT reagent Kit (RR047A, Takara Bio Inc., China). The expression levels of TNF-α and IL-1β were detected using TB Green Premix Ex Taq II (RR820A, Takara Bio Inc., China). GAPDH was regarded as the internal control. The primer sequences are provided in Table 2.

### 2.12. Statistical Data Analysis

IBM SPSS 26.0 software (IBM, Corp., USA) and GraphPad Prism 8 software (GraphPad, USA) were employed to analyze the data and draw the statistical charts. The data were displayed as mean ± standard error of the mean (SEM). One-way analysis of variance (ANOVA) was adopted to compare the results, followed by Tukey′s method or Dunnett T3 method. A *p*-value < 0.05 was considered statistically significant.

## 3. Results

### 3.1. Network Pharmacology Analysis to Unveil the Mechanism of PF against SIMI

#### 3.1.1. Identification of PF-Related and SIMI-Related Targets

**Figure 2:**
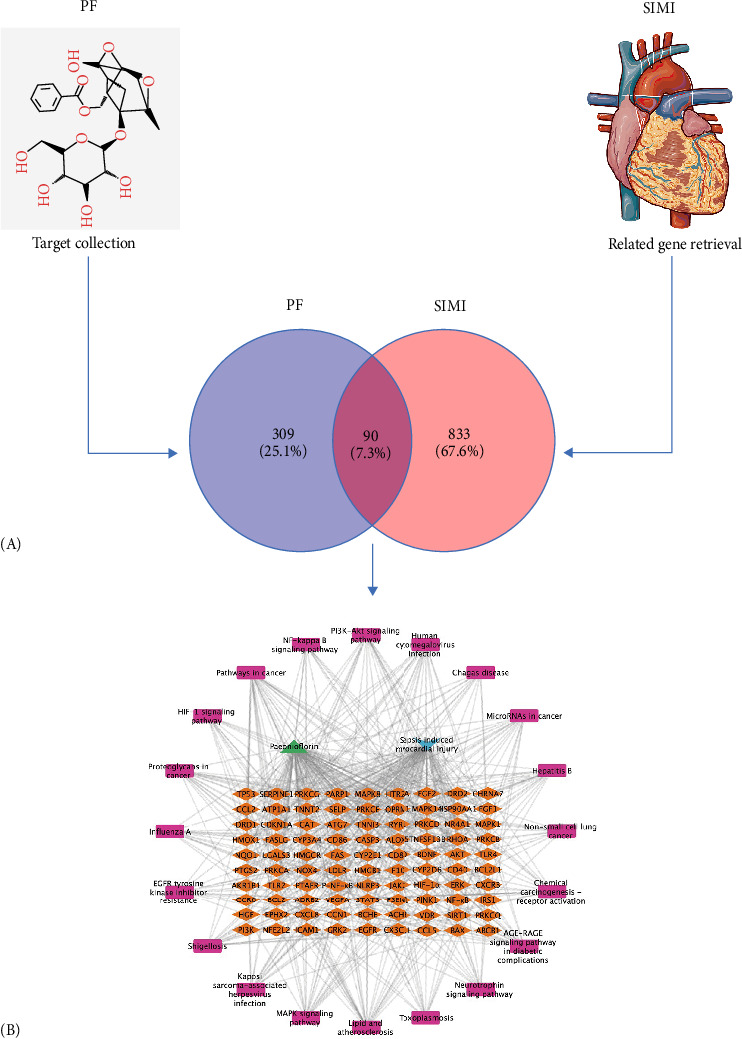
The PF-target-SIMI network construction. (A) Identification of intersection targets between PF and SIMI. (B) Construction of a “PF-SIMI-Intersection targets-signaling pathway” network.

A total of 399 potential targets of PF were identified from various databases and bibliographic retrieval. Similarly, 923 targets associated with SIMI were retrieved from the GeneCard, OMIM, and DrugBank databases, with relevance scores over 4.211. Upon intersecting these datasets, 90 overlapping targets were pinpointed and visualized in the Venn diagram (Figure 2A). These 90 overlapping targets were selected for subsequent enrichment analysis and were deemed the action targets of PF in treating SIMI. These results were further utilized to construct a “PF-SIMI-Intersection targets-signaling pathway” network using Cytoscape software 3.10.3 (Figure 2B).

#### 3.1.2. PPI Analysis of the Targets of PF on SIMI

**Figure 3:**
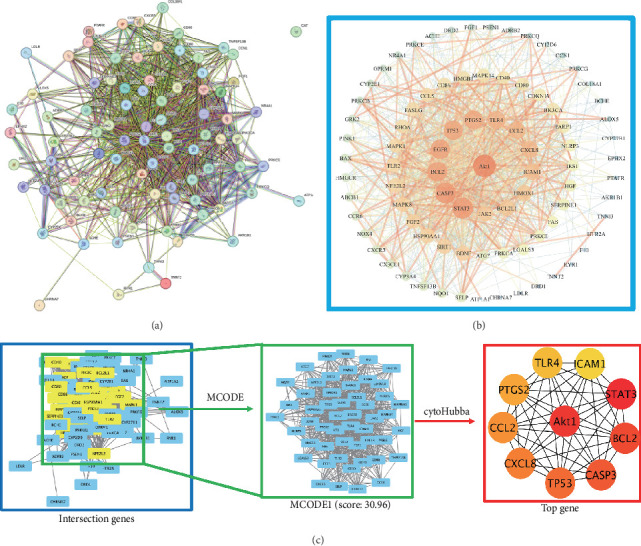
PPI analysis of the targets of PF against SIMI. (A) The PPI network was obtained from STRING database. (B) The intersecting targets were visualized by Cytoscape. (C) The significant module was obtained from the PIN by MCODE method with a score >4.0; red represents the higher degree and yellow represents the lower degree.

To elucidate the core targets of PF in treating SIMI, the 90 intersecting target genes were subjected to PPI network analysis using the STRING database (score > 0.7) (Figure 3A) and visualized with Cytoscape. The resultant network highlighted several key targets, including Akt1, STAT3, CASP3, BCL2, EGFR, TP53, PTGS2, and TLR4 (Figure 3B), which are implicated in signal transduction, cell proliferation/differentiation, apoptosis, and inflammatory responses, respectively. Further the biological functional clustering using MCODE method analysis identified a significant module with a score exceeding 30, comprising 10 genes: Akt1, STAT3, CASP3, BCL2, TP53, PTGS2, CXCL8, TLR4, CCL2, and ICAM1 (Figure 3C). Within the gene network, Akt1, STAT3 [15], CXCL8 [16], PTGS2, TLR4, CCL2, and ICAM1 are closely associated with inflammation and immunity [17]. Additionally, CASP3, BCL2, and TP53 are related to apoptosis [18]. These findings suggest that PF may improve SIMI by inhibiting inflammation, regulating immunity, and reducing cardiomyocyte apoptosis.

#### 3.1.3. GO and KEGG Enrichment Analysis

**Figure 4:**
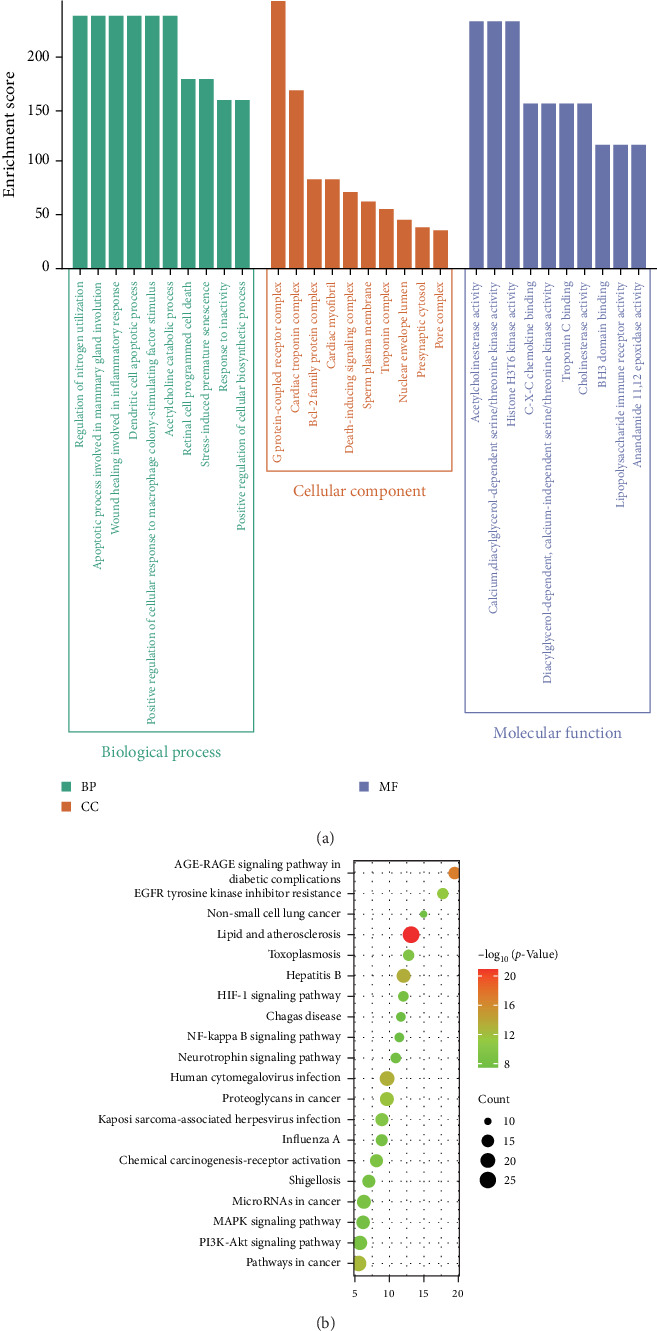
GO and KEGG enrichment analysis of target genes of PF against SIMI. (A) Enriched GO terms for biological process (BP), cellular component (CC), and molecular function (MF) of potential anti-SIMI targets of PF. (B) Enriched KEGG pathways of potential anti-SIMI targets of PF.

The GO functional enrichment analysis showed that 90 PF-regulated genes play crucial roles in tissue repair, involved in inflammation, apoptosis, cell death, and immunity. These processes include wound repair involved in inflammatory response, apoptotic process involved in mammary gland involution, dendritic cell apoptotic process, retinal cell programed cell death, stress-induced premature senescence, response to inactivity, positive regulation of cellular response to macrophage colony-stimulating factor stimulus, regulation of nitrogen utilization, et cetera. (Figure 4A). This suggests that PF may promote the myocardial repair and reduce inflammation through these biological pathways. KEGG enrichment analysis further illustrated that the core signaling pathways involved in inflammation include the NF-κB signaling pathway, HIF-1 signaling pathway, MAPK signaling pathway, and PI3K-Akt signaling pathway (Figure 4B). The results indicate that PF may protect the heart from damage by regulating these key inflammatory signaling pathways.

### 3.2. Validation of Specific Target-PF Interactions by Molecular Docking

**Figure 5:**
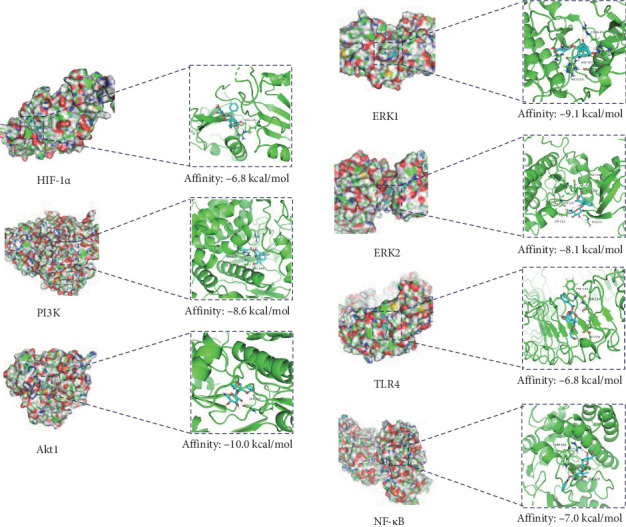
Molecular docking of PF with PI3K, Akt1, ERK1, ERK2, HIF-1α, TLR4, and NF-κB.

PPI and MCODE analyses identified Akt1 and TLR4 as key targets in PF's anti-SIMI effects, while KEGG analysis highlighted several inflammation-related signaling pathways, including NF-κB, HIF-1, MAPK, and PI3K-Akt pathways. To further explore these interactions, we performed molecular docking studies to assess the binding affinities of PF with key inflammatory signaling molecules, such as PI3K, Akt1, ERK1, ERK2, HIF-1α, TLR4, and NF-κB. The results demonstrated strong binding affinities between PF and these targets, with binding energies significantly below –(6–10) kcal/mol, especially, the binding energy between PF and Akt1 was −10 kcal/mol suggesting the strong binding ability (Figure 5). These findings suggest that PF may effectively resolve inflammation in SIMI by regulating inflammation-related targets (PI3K, Akt1, ERK1, ERK2, HIF-1α, TLR4, and NF-κB).

### 3.3. PF Ameliorates LPS-Induced Cardiac Dysfunction in Rats

We assessed cardiac function using ECG and echocardiograph. ECG lead II recordings showed a significant elevation of the ST-segment in the LPS group, which was inhibited by PF and Dex pretreatment (Figure 1A). Echocardiographic analysis revealed that EF, FS, and CO were decreased in the LPS group compared to the control group, while PF and Dex prevented these decreases (Figure 1). Moreover, LVPWs and LVPWd were thinner in the LPS group than in the control group, while PF and Dex restored the reduction of LVPWs and LVPWd (Figure 1F, G). At the same time, LVAWd was thicker in the LPS group than in the control group, and PF and Dex did not prevent this change (Figure 1H). There were no differences in LVAWs, LVIDs, and LVIDd observed among the four groups (Figure 1). These data indicate that PF effectively ameliorates LPS-induced cardiac dysfunction in rats.

### 3.4. PF Mitigates LPS-Induced Acute Myocardial Injury

**Figure 6:**
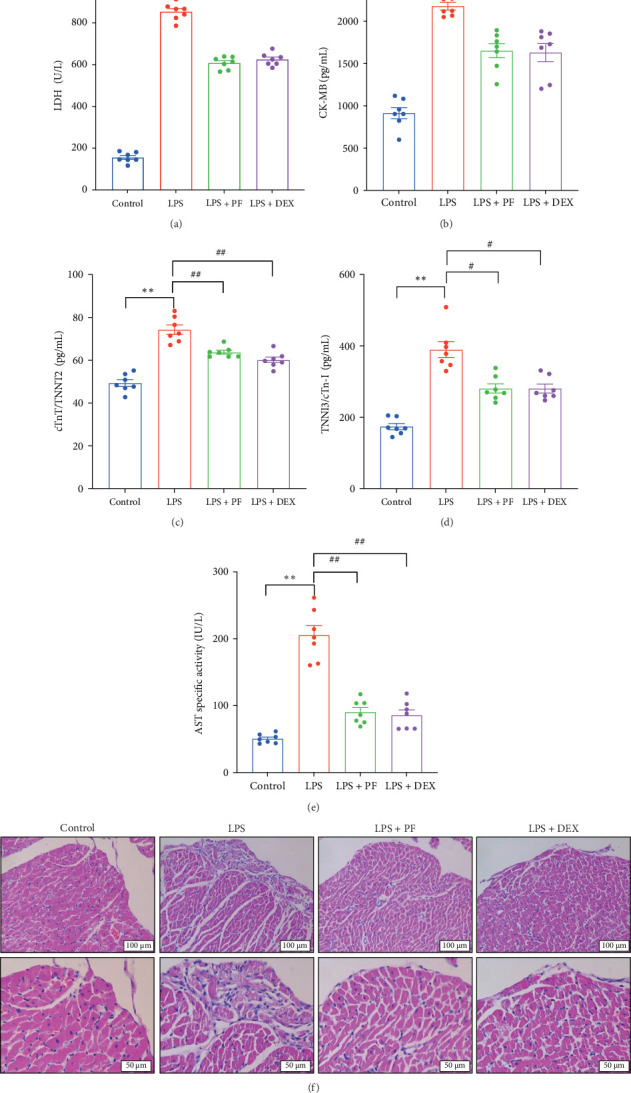
PF mitigates LPS-induced acute myocardial injury. (A–E) The levels of serum LDH, CK-MB, cTnT/TNNT2, TNNI3/cTn-I, and AST. (F) HE staining of left ventricular tissue in each group. Scale bars = 100 μm or 50 μm. Significance: *⁣*^*∗*^*p* < 0.05, *⁣*^*∗∗*^*p* < 0.01 vs. the control group; ^#^*p* < 0.05, ^##^*p* < 0.01 vs. the LPS group.

Serum levels of myocardial injury markers, including LDH, CK-MB, cTnT/TNNT2, TNNI3/cTn-I, and AST, were significantly elevated in the LPS group compared to the control group, the results are as follows: LDH (854.39 ± 15.44 U/L vs. 155.57 ± 9.22 U/L, *p* < 0.01), CK-MB (2180.84 ± 39.99 pg/mL vs. 914.79 ± 56.49 pg/mL, *p* < 0.01), cTnT/TNNT2 (74.28 ± 2.24 pg/mL vs. 49.36 ± 1.59 pg/mL, *p* < 0.01), TNNI3/cTn-I (389.61 ± 22.50 pg/mL vs. 174.02 ± 8.58 pg/mL, *p* < 0.01), and AST (205.29 ± 14.33 IU/L vs. 50.62 ± 2.63 IU/L, *p* < 0.01) (Figure 6). However, compared with the LPS group, PF and Dex pretreatment significantly reduced these elevations, as evidenced by the decreased levels of LDH (608.93 ± 11.09 U/L vs. 854.39 ± 15.44 U/L, *p* < 0.01; 625.59 ± 11.48 U/L vs. 854.39 ± 15.44 U/L, *p* < 0.01), CK-MB (1652.75 ± 72.57 pg/mL vs. 2180.84 ± 39.99 pg/mL, *p* < 0.01; 1631.29 ± 93.63 pg/mL vs. 2180.84 ± 39.99 pg/mL, *p* < 0.05), cTnT/TNNT2 (63.75 ± 0.95 pg/mL vs. 74.28 ± 2.24 pg/mL, *p* < 0.01; 60.10 ± 1.37 pg/mL vs. 74.28 ± 2.24 pg/mL, *p* < 0.01), TNNI3/cTn-I (280.82 ± 12.86 pg/mL vs. 389.61 ± 22.50 pg/mL, *p* < 0.01; 280.73 ± 12.31 pg/mL vs. 389.61 ± 22.50 pg/mL, *p* < 0.01), and AST (90.35 ± 6.82 IU/L vs. 205.29 ± 14.33 IU/L, *p* < 0.01; 85.70 ± 7.90 IU/L vs. 205.29 ± 14.33 IU/L, *p* < 0.01) (Figure 6).

HE staining was used to assess the histopathological changes in hearts from different groups. The LPS group appeared myocardial structure disturbances, loose connective tissue arrangement, wavy myocardial fiber, eosinophilic changes, and even rupture, infiltration of inflammatory cells, and nuclear deformation, while pretreated with PF and Dex reversed these adverse changes (Figure 6F). These evidences underscore the protection of PF against acute myocardial injury induced by LPS.

### 3.5. PF Alleviates LPS-Increased Cardiac Inflammatory Response

**Figure 7:**
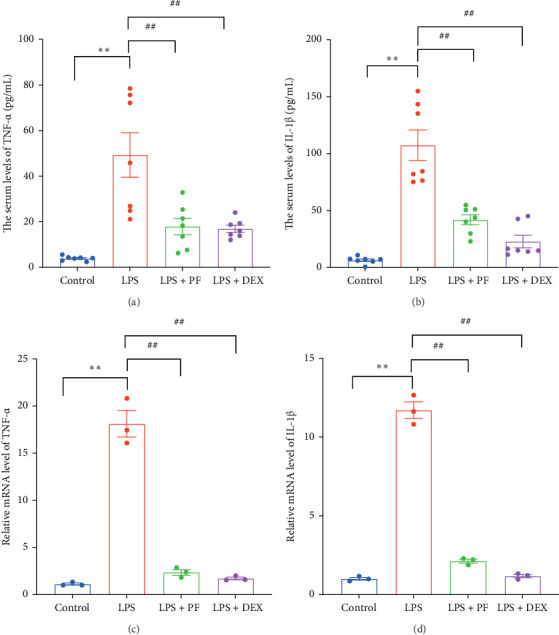
PF alleviates LPS-increased cardiac inflammatory response. (A, B) The levels of serum TNF-α and IL-1β. (C, D) The mRNA levels of TNF-α and IL-1β were detected by qRT-PCR. Significance: *⁣*^*∗*^*p* < 0.05, *⁣*^*∗∗*^*p* < 0.01 vs. the control group; ^#^*p* < 0.05, ^##^*p* < 0.01 vs. the LPS group.

Since excessive inflammation plays a vital role in the pathogenesis of sepsis-associated cardiac abnormalities, we measured the levels of inflammatory cytokines TNF-α and IL-1β. ELISA analysis demonstrated that the levels of TNF-α and IL-1β were elevated in the LPS group compared to the control group, the results were as follows: TNF-α (49.31 ± 9.74 pg/mL vs. 3.92 ± 0.38 pg/mL, *p* < 0.01) and IL-1β (107.40 ± 13.35 pg/mL vs. 6.49 ± 1.16 pg/mL, *p* < 0.01). However, PF and Dex decreased these elevations as illustrating with TNF-α (17.91 ± 3.63 vs. 49.31 ± 9.74 pg/mL, *p* < 0.01; 16.90 ± 1.54 vs. 49.31 ± 9.74 pg/mL, *p* < 0.01) and IL-1β (41.98 ± 4.46 pg/mL vs. 107.40 ± 13.35 pg/mL, *p* < 0.01; 22.85 ± 5.50 pg/mL vs. 107.40 ± 13.35 pg/mL, *p* < 0.01) (Figure 7A, B). qRT-PCR analysis further illustrated that the levels of TNF-α and IL-1β were upregulated by LPS-administration, while PF and Dex reduced these upregulations of TNF-α and IL-1β in cardiac tissue (Figure 7C, D). These results reconfirm the PF's anti-inflammatory effects through the inhibition of inflammatory cytokine release.

### 3.6. PF Blocks TLR4/MyD88/NF-κB Signaling in LPS-Induced SIMI

**Figure 8:**
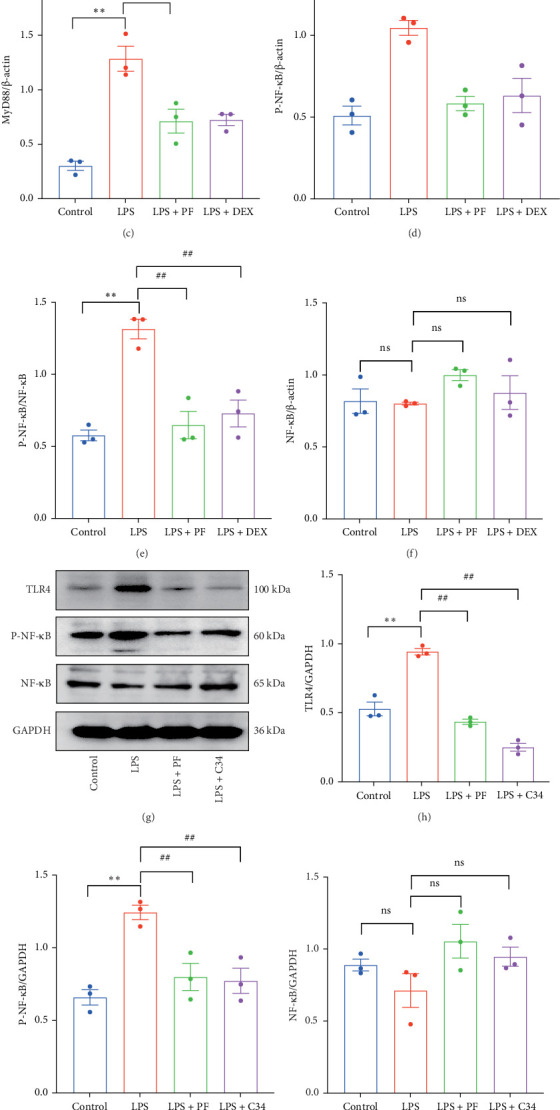
PF blocks TLR4/MyD88/NF-κB signaling in LPS-induced SIMI. (A) Western blot detection of TLR4, MyD88, P-NF-κB, and NF-κB. (B–F) Bar graphs show the relative expression of TLR4, Myd88, P-NF-κB, NF-κB, and the ratio of p-NF-κB/NF-κB as analyzed by western blot. β-actin was used as an internal control (*n* = 3). (G) Western blot detection of TLR4, P-NF-κB, and NF-κB. (H–K) Bar graphs show the relative expression of TLR4, p-NF-κB, NF-κB, and the ratio of p-NF-κB/NF-κB as analyzed by western blot. GAPDH was used as an internal control (*n* = 3). Data were shown as mean ± SEM. Significance: *⁣*^*∗*^*p* < 0.05, *⁣*^*∗∗*^*p* < 0.01 vs. the control group; ^#^*p* < 0.05, ^##^*p* < 0.01 vs. the LPS group.

Both the network analysis and the existing reports have shown that TLR4/MyD88/NF-κB inflammation signaling is an important contributor to LPS-induced inflammation and organ injury [14]. In the study, we detected whether TLR4/MyD88/NF-κB signaling is activated following LPS infusion. Western blot analysis demonstrated that LPS upregulated the expression of TLR4, MyD88, and P-NF-κB (Figure 8), while PF and Dex blocked the activation of TLR4/MyD88/NF-κB signaling, as evidenced by the reduced expression of TLR4, MyD88 and P-NF-κB induced by LPS (Figure 8A–F). Additionally, PF exhibited an inhibitory effect on TLR4/NF-κB signaling similar to that of the TLR4 inhibitor C34 (Figure 8). These data illustrate that PF can effectively block TLR4/MyD88/NF-κB signaling activated by LPS infusion.

### 3.7. PF Obstructs PI3K/Akt/ERK-Mediated HIF-1α in LPS-Induced SIMI

**Figure 9:**
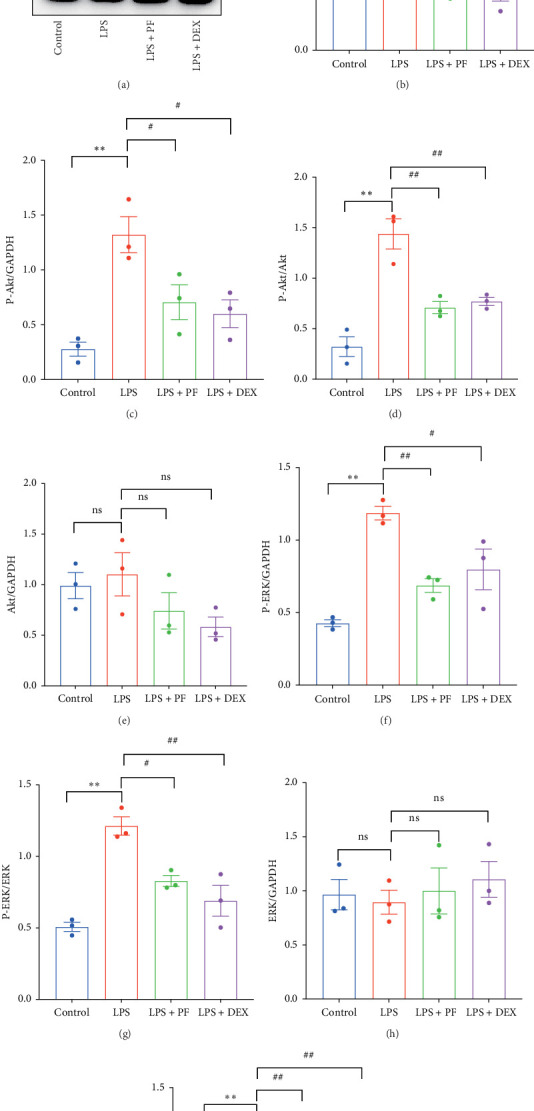
PF obstructs PI3K/Akt/ERK-mediated HIF-1α in LPS-induced SIMI. (A) Western blot detection of PI3K, P-Akt, Akt, P-ERK, ERK, and HIF-1α. (B–I) Bar graphs show the relative expression of PI3K, P-Akt, Akt, P-ERK, ERK, HIF-1α, and the ratio of P-Akt/Akt and P-ERK/ERK as analyzed by western blot. GAPDH was used as an internal control (*n* = 3). Data were shown as mean ± SEM. Significance: *⁣*^*∗*^*p* < 0.05, *⁣*^*∗∗*^*p* < 0.01 vs. the control group; ^#^*p* < 0.05, ^##^*p* < 0.01 vs. the LPS group.

PI3K/Akt/ERK signaling is implicated in inflammation and can mediate the expression of HIF-1α [19, 20]. Moreover, PF exhibits strikingly high binding abilities with targets, such as PI3K, Akt1, ERK1, ERK2, and HIF-1α. Western blot was used to test the expression of PI3K, Akt, ERK, and HIF-1α and the levels of P-Akt and P-ERK. The results showed that LPS challenge observably increased the expression of PI3K and HIF-1α and the phosphorylation levels of Akt and ERK (Figure 9). However, PF and Dex pretreatment exhibited markedly downregulation of the expression of PI3K and HIF-1α and the phosphorylation levels of Akt and ERK (Figure 9). These data demonstrate that PF can effectively obstruct PI3K/Akt/ERK-mediated HIF-1α in LPS-induced SIMI.

## 4. Discussion

Sepsis is a life-threatening condition characterized by a systemic inflammatory response to infection, often leading to multiple organ dysfunction, including severe cardiac complications. Cardiac dysfunction is a common and severe complication caused by sepsis with the fatality rate of 70%–90% [21]. Previous studies have proved that PF can inhibit TNF-α and IL-1β release and improve cardiac systolic function in mice challenged with LPS [9, 22]. A similar effect was exhibited in this study; we found that PF significantly decreased the release of TNF-α and IL-1β in the serum of LPS-administrated rats (Figure 7A, B) and prevented cardiac inflammation, as illustrated by the reduction of TNF-α and IL-1β in cardiac tissue (Figure 7C, D). Furthermore, PF alleviated acute myocardial injury, as evidenced by mitigated levels of the myocardial injury markers as such LDH, CK-MB, cTnT/TNNT2, TNNI3/cTn-I, and AST, and improved cardiac pathological changes (Figure 6). Ultimately, PF significantly improved LPS-induced cardiac dysfunction, as evidenced by the decreased elevation of ST-segment in ECG lead II and the prevention of reductions in EF, FS, CO, LVPWs, and LVPWd (Figure 1).

Promising studies have integrated network pharmacology, molecular docking, and experimental validation to explore the molecular mechanism underlying drug therapeutic effects. In the study, we applied network pharmacology to establish a comprehensive “drug-target-gene” interaction network, revealing how PF affects inflammation in septic myocardial injury networks. We identified 90 overlapping targets between PF and SIMI (Figure 2), with significant modules and key targets, such as Akt1, STAT3, CASP3, BCL2, TP53, PTGS2, CXCL8, TLR4, CCL2, and ICAM1 (Figure 3). These targets are closely related to inflammation, apoptosis, and immunity [15–18]. By modulating these targets, PF can exert broad anti-inflammatory and cardioprotective effects. GO enrichment analysis highlighted their significant involvement in biological processes, cellular composition, and molecular functions, with a particular emphasis on tissue repair involved in inflammation, apoptosis, cell death, and immunity (Figure 4A). These findings suggest that PF can inhibit inflammation in the heart, thereby mitigating myocardial injury associated with sepsis [22].

KEGG enrichment analysis identified key signaling pathways associated with SIMI, including inflammation signaling (NF-κB signaling pathway, HIF-1 signaling pathway, MAPK signaling pathway, and PI3K-Akt signaling pathway) and various infections (Figure 4B). These pathways play a vital role in inflammatory response and cardiac injury in sepsis. Previous studies have also revealed that the related role of PI3K/Akt [23], ERK [24], TLR4/NF-κB [25], and HIF-1α [26] involved in sepsis-associated inflammation and cardiac injury. Nowadays, molecular docking has also verified the strong binding affinity between PF and core targets, such as PI3K, Akt1, ERK1, ERK2, TLR4, NF-κB, and HIF-1α (Figure 5). This evidence supports the notion that PF is a promising therapeutic agent for improving SIMI by regulating inflammation-related signaling pathways, such as PI3K/Akt1, ERK1/2, TLR4/NF-κB, and HIF-1α.

Experimental validation further elucidated the effect mechanism of PF on SIMI. Toll-like receptors (TLRs) are commonly activated receptors in sepsis, leading to the activation of NF-κB and the subsequent release of inflammatory factors, such as TNF-α, IL-1β, IL-6, and IL-18 [6]. In LPS-caused left ventricular remodeling and dysfunction, TLR4/NF-κB/NLRP3 signaling activation promotes inflammatory responses and cardiomyocytes apoptosis [27]. We also verified the activation of TLR4/MyD88/NF-κB signaling in cardiac tissue following LPS injection, which was effectively blocked by PF, as evidenced by reduced expression of TLR4 and MyD88 expression and decreased phosphorylation level of NF-κB in cardiac tissue (Figure 8). Additionally, PF exhibited a similar inhibitory effect as the TLR4 inhibitor C34 on TLR4/NF-κB signaling (Figure 8). These findings suggest that PF may act as a potent inhibitor of TLR4/NF-κB signaling.

Promising experiments have showed that HIF-1α plays an important role in the inflammatory regulation and organ dysfunction associated with sepsis [26]. HIF-1α can promote TLR4 expression by enhancing the transcriptional activity of the TLR4 promoter [28]. In LPS-induced SIMI, we found that PF significantly decreased the expression of HIF-1α in cardiac tissue (Figure 9). As such, it is likely that inhibiting HIF-1α is a pivotal target for preventing the inflammation regulated by TLR4/NF-κB pathway. Therefore, PF may be as an inhibitor of the HIF-1α to block TLR4/NF-κB signaling.

Existing research has demonstrated that PI3K/Akt signaling pathway is involved in HIF-1α regulation in sepsis [29], and HIF-1α can be mediated by PI3K/Akt/ERK pathway in retinopathy of prematurity [20]. Therefore, the expression of HIF-1α may be reduced by preventing the activation of PI3K/Akt/ERK signaling pathway. In our study, we clarified that PF restrained the activation of PI3K/Akt/ERK signaling pathway, as illustrated by reduced expression of PI3K and decreased phosphorylation levels of Akt and ERK1/2 (Figure 9). These results suggest that PF may impede the expression of HIF-1α by inhibiting PI3K/Akt/ERK signaling, thereby downregulating the transcriptional activity of TLR4 and other inflammation-related genes. This cross-talk between different signaling pathways provides a more comprehensive explanation of how PF exerts its anti-inflammatory and cardioprotective effects.

In this study, we identified the molecular targets and signaling pathways of PF intervention in inflammation to improve SIMI by means of network pharmacology and molecular docking. These predicted targets and signaling pathways were further affirmed through experimental studies using LPS-induced sepsis model, which provided a theoretical basis for exploring the mechanism of PF on SIMI. However, there are still some questions that require further in-depth study in the future. For example, although network pharmacology and molecular docking provided valuable insights into the potential targets and mechanisms of PF in treating SIMI, these computational predictions need to be further validated by in vitro experiments. Moreover, the cross-talk between different signaling pathways needs to be confirmed through knockdown or overexpression experiments.

## 5. Conclusions

**Figure 10:**
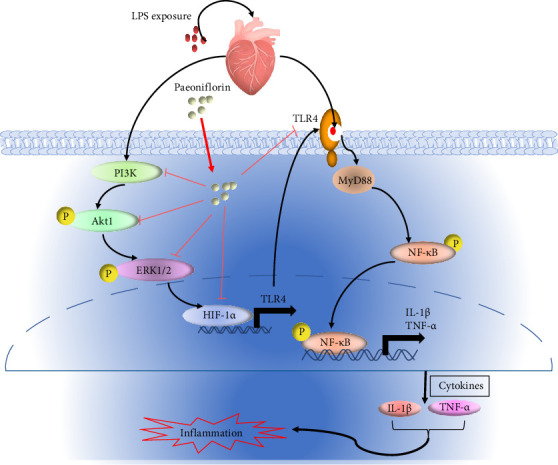
The schematic diagram for PF anti-SIMI by inhibiting PI3K/Akt/ERK-mediated HIF-1α and TLR4/MyD88/NF-κB.

We performed a network analysis of PF and SIMI to predict the pathway and biological functions of network pharmacology. The data showed that 90 targets were closely associated with PF's effect in treating SIMI by regulating inflammation through the NF-κB, HIF-1α, ERK, and PI3K/Akt signaling pathways. Molecular docking analysis validated the strong binding affinity between the predicted target candidates (PI3K, Akt, ERK1, ERK2, TLR4, NF-κB, and HIF-1 α) and PF. Furthermore, we verified the therapeutic role of PF in treating SIMI by conducting an LPS-challenged sepsis model. This model indicated a decrease in proinflammatory factors and myocardial injury markers by PF pretreatment in cardiac tissue. Finally, we validated the inhibitory role of PF on SIMI by down-regulating the expression of HIF-1α as well as by regulating theTLR4/NF-κB. Additionally, PF impeded the PI3K/Akt/ERK signaling pathway, which is closely related to the expression of HIF-1α. This research provides a more thorough evaluation and understanding of PF's role in treating SIMI and new opportunities for developing PF-based products (Figure 10).

## Figures and Tables

**Table 1:** Reagents and antibodies.

Name	Company	Product number
Lactate dehydrogenase (LDH) activity assay kit	Elabscience biotechnology Co., Ltd (China)	E-BC-K046-M
Rat CK-MB (creatine kinase MB isoenzyme) ELISA kit	Elabscience biotechnology Co., Ltd (China)	E-EL-R1327c
Rat cTnT/TNNT2 (Troponin T type 2, cardiac) ELISA kit	Elabscience biotechnology Co., Ltd (China)	E-EL-R0151c
Rat TNNI3/cTn-I (Troponin I type 3, cardiac) ELISA kit	Elabscience biotechnology Co., Ltd (China)	E-EL-R1253c
Aspartate aminotransferase (AST) activity assay kit (colorimetric)	Elabscience biotechnology Co., Ltd (China)	E-BC-K236-M
Rat TNF-α (tumor necrosis factor alpha) ELISA kit	Elabscience biotechnology Co., Ltd (China)	E-EL-R2856c
Rat IL-1β (interleukin 1 beta) ELISA kit	Elabscience biotechnology Co., Ltd (China)	E-EL-R0012c
H&E staining kits	Servicebio (China)	G1076-500ML
BCA protein quantification kit	Solarbio (China)	PC0020
The total RNA Kit II	Omega (USA)	R6934-01
PrimeScript RT reagent Kit	Takara bio Inc. (China)	RR047A
TB Green Premix Ex Taq II	Takara bio Inc. (China)	RR820A
HIF-1α rabbit mAb (D2U3T)	Cell signaling technology (USA)	14179
TLR4 monoclonal antibody	Proteintech (China)	66350-1-Ig
MyD88 rabbit mAb (D80F5)	Cell signaling technology (USA)	4283
NF-κB-p65 polyclonal antibody (phospho Ser536)	Immunoway (USA)	YP0191
NF-κB p65 XP rabbit mAb (D14E12)	Cell signaling technology (USA)	8242
PI3K recombinant rabbit monoclonal antibody (SU04-07)	Hangzhou HuaAn biotechnology Co., Ltd (China)	ET1608-70
AKT1/2/3 recombinant rabbit monoclonal antibody (ST48-09)	Hangzhou HuaAn biotechnology Co., Ltd (China)	ET1609-51
Phospho-AKT (S473) recombinant rabbit monoclonal antibody (SY28-05)	Hangzhou HuaAn biotechnology Co., Ltd (China)	ET1607-73
ERK1/2 recombinant rabbit monoclonal antibody (SA43-03)	Hangzhou HuaAn biotechnology Co., Ltd (China)	ET1601-29
Phospho-Erk1 (T202 + Y204) + Erk2 (T185 + Y187) recombinant rabbit monoclonal antibody (SC58-01)	Hangzhou HuaAn biotechnology Co., Ltd (China)	ET1610-13
GAPDH antibody	Proteintech (China)	6004-1-Ig
β-actin antibody	Proteintech (China)	66009-1-Ig
HRP-conjugated goat anti-mouse antibody	ZSGB (China)	ZB-2305
HRP-conjugated goat anti-rabbit antibody	ZSGB (China)	ZB-2301

**Table 2:** Primer sequences of selected genes.

Name	Sequence 5′–3′
GAPDH	
Forward	5′-CAGGGCTGCCTTCTCTTGTG-3′
Reverse	5′-AACTTGCCGTGGGTAGAGTC-3′
TNF-α	
Forward	5′-CCACCACGCTCTTCTGTCTACTG-3′
Reverse	5′-TGGGCTACGGGCTTGTCACT-3′
IL-1β	
Forward	5′-GTGGCAGCTACCTATGTCTTGC-3′
Reverse	5′-CCACTTGTTGGCTTATGTTCTGT-3′

## Data Availability

The data that support the findings of this study are available from the corresponding author upon reasonable request.
